# Interneuronal Mechanism for Tinbergen’s Hierarchical Model of Behavioral Choice

**DOI:** 10.1016/j.cub.2014.09.024

**Published:** 2014-09-22

**Authors:** Zsolt Pirger, Michael Crossley, Zita László, Souvik Naskar, György Kemenes, Michael O’Shea, Paul R. Benjamin, Ildikó Kemenes

(Current Biology *24*, 2018–2024; September 8, 2014)

A reader has brought to our attention a labeling error in Figure 2 of this manuscript as originally published online and in print: the two arrows pointing upward under panels D4 and D5 should have been labeled “touch.” This error has now been corrected in Figure 2 of the article online. The authors apologize for any confusion this error may have caused.

