# Supplementary material for: Interneuronal Mechanism for Tinbergen’s Hierarchical Model of Behavioral Choice
Source: Curr Biol. 2014 Sep 8;24(17):2018–24. doi: 10.1016/j.cub.2014.07.044 (PMC4159561; doi:10.1016/j.cub.2014.07.044)
Supplement: Document S1. Supplemental Experimental Procedures and Figures S1 and S2 [file mmc1.pdf]

Current Biology, Volume *24*

Supplemental Information

Interneuronal Mechanism for Tinbergen's Hierarchical Model of Behavioral Choice

Zsolt Pirger, Michael Crossley, Zita László, Souvik Naskar, György Kemenes, Michael O'Shea, Paul R. Benjamin, and Ildikó Kemenes

Supplemental figures

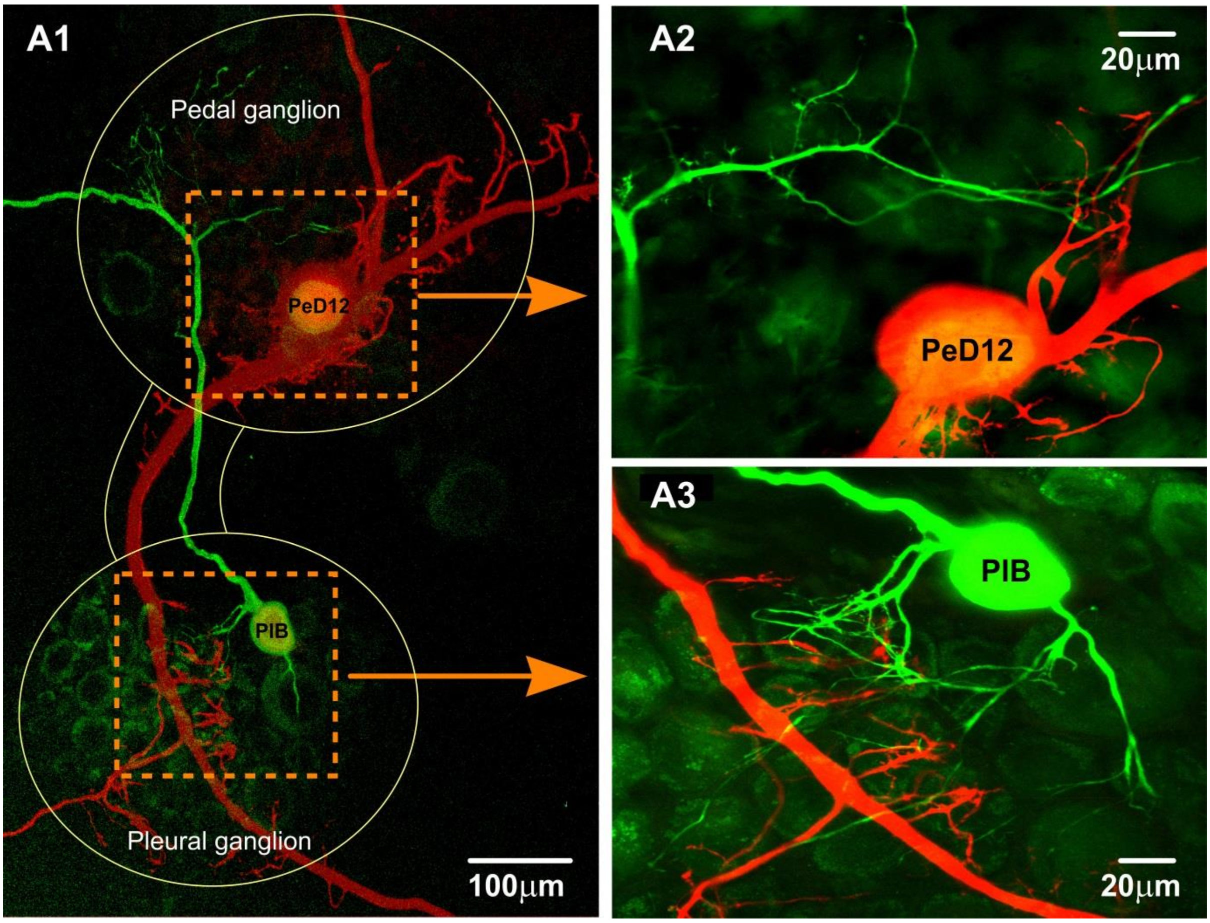

Figure S1. Morphology and Possible Sites of Synaptic Connections between the PlB and PeD12 Neurons.

(A1) Arborization and likely anatomical locations (dashed rectangles) of synaptic connections between the neurites of PeD12 and PlB, revealed by the injection of Alexa Red into PeD12 and Lucifer Yellow into PlB. (A2, A3). Two potential sites of the synaptic interactions recorded in the experiments shown in Figure 3A, B.

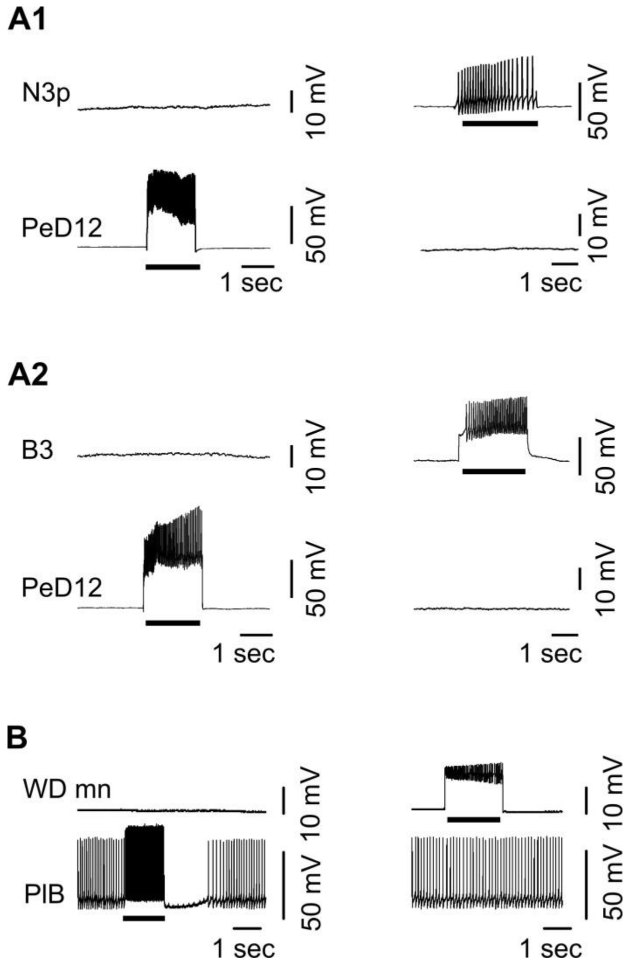

Figure S2. Lack of Direct Synaptic Connections between the Interneurons and Motoneurons that Control the Two Behavioral Circuits

There are no reciprocal synaptic connections between the PeD12 neuron and the central pattern generator interneuron, N3p (A1) and motoneurons (A2) of the feeding network (see Figure 2A, D). The experiments were carried out in Hi-Di saline to block any polysynaptic effects of PeD12 on feeding neurons. (B) There are no reciprocal synaptic connections between PIB and withdrawal motoneurons (WD mn) (Figure 2A, C).

Supplemental Data

Anatomical Evidence for Monosynaptic Connection between PeD12 and PlB

Injection of flourescent dyes revealed the likely location of the synaptic connection between PeD12 and PlB. Figure S1A shows the axonal projections of the two cells within the CNS ganglia. There is close intertwining of fine neuritic processes between PeD12 and PlB at two sites, one close to the PeD12 cell body in the pedal ganglion and the other close to the PlB cell body in the pleural ganglion (boxed areas). Viewing these sites at higher magnification shows the close apposition of the fine branching processes at these two locations, in the pedal (Figure SA2) and pleural (Figure SA1) ganglia neuropile. This combined with the electrophysiological data presented in Figure 3B suggest that the two interneurons are monosynaptically connected.

Lack of Direct Synaptic connections between PeD12 and PlB and Neurons of the Feeding and Withdrawal Response Networks

No reciprocal synaptic connections were found between PeD12 and central pattern generator interneurons such as the N3p (Figure S2A1) and motoneurons (Figure S2A2) of the feeding network (n = 12). No post-synaptic potentials or spike responses occurred as a result of current-evoked bursts of spikes in either cell types. These experiments were carried out in Hi-Di saline to prevent polysynaptic responses on feeding neurons due to the indirect PeD12-PlB pathway. There were a similar lack of reciprocal post-synaptic responses when PlB was recorded with motoneurons of the withdrawal response network (Figure S2B)(n= 10).

Supplemental Experimental Procedures

Animal Maintenance

Animals were kept in groups in large holding tanks containing copper-free water at 20°C on a 12:12h light-dark regime. The animals were fed lettuce three times and a vegetable based fish food (Tetra-Phyll; TETRA Werke, Melle, Germany) twice a week except before starting an experiment, when they were not fed for two days.

Supplemental Behavioral Procedures

To activate fictive feeding in the semi-intact preparations, sucrose (0.02 mM in normal saline) was applied to the lips via a computer controlled gravity-fed perfusion system. A mechanical probe connected to a DC power supply was used for tactile stimulation of the lips allowing for exact timing of touch stimulation. The stimulus, reliably generated whole body withdrawal responses monitored both on the columellar muscle and on the withdrawal motoneurons. The threshold level necessary for the whole-body withdrawal responses were carefully tested in both behavioral and semi-intact preparations to make sure we were dealing with the all–or–nothing responses rather than local graded responses. Experiments began 10 min after testing for a threshold. Preliminary experiments showed that keeping this time interval produces no change in the response. The buccal and columellar muscles were attached to force transducers (WPI FORT10g, World Precision Instruments, Incorporation, Sarasota, USA). Muscle recordings were made by connecting, the force transducers through a Digidata 1320A interface (Axon Instruments, Union City, CA, USA) to a PC.

Supplemental Electrophysiology Procedures

Preparations were dissected and recorded in a Sylgard-lined chamber containing normal snail saline (NS, 50 mM NaCl, 1.6 mM KCl, 3.5 mM $CaCl_2$, 2.0 mM $MgCl_2$, 10 mM HEPES, pH 7.9). The outer layer of the thick connective tissue sheath was removed mechanically from the dorsal surface of ganglia and the inner layers were softened by 1% protease treatment (Sigma XIV, Sigma) for 2 min. Synaptic connections between target cells were investigated in Hi-Lo EGTA saline (35.0 mM NaCl, 1.6 mM KCl, 18.0 mM $MgCl_2$, 10 mM HEPES, 2.0 mM EGTA, pH 7.9.) and Hi-Di saline (35.0 mM NaCl, 2 mM KCl, 14.0 mM $CaCl_2$, 8.0 mM $MgCl_2$, 10 mM HEPES, pH 7.9)[S1].

Intracellular recordings were performed under a fluorescence stereomicroscope (Leica MZ FLIII, Switzerland). AxoClamp 2B (Axon Instruments, Union City, CA, USA) and NeuroLog D.C. (Digitimer Ltd., UK) amplifiers were used to monitor the electrical activity of cells. Membrane potential manipulation was carried out by current injection through the recording electrode or by use of separate current-injecting and voltage–recording electrodes when more accurate recordings of membrane potential was required. Microelectrodes were pulled from borosilicate glass pipettes (GC200F-15, Harvard Apparatus, UK) with Narishige (Narishige Scientific Instrument Laboratory, Japan) vertical puller to a 18–24 MΩ tip resistance when filled with 4 M potassium acetate. For data acquisition and protocols, the amplifiers were connected via a DigiData 1320A interface (Axon Instruments, Union City, CA, USA) to a PC supplied with pClamp8.2 software (Axon Instruments, Union City, CA, USA). The recorded traces were analysed by OriginLab Corporation Origin8.5 software.

Identification of neurons

The B3 feeding motoneuron of the buccal ganglia was mainly used to monitor the CPG-driven fictive feeding rhythm. B4 feeding motoneurons were also routinely recorded to confirm the occurrence of feeding cycles. Both types of neuron can be identified by size, location and characteristic feeding activity [S2]. To monitor electrical activity underlying whole-body withdrawal, motoneurons of the cerebral A cluster, G cluster neurons of the pedal ganglion and the single DLM of left parietal were recorded, often in combination. These neurons can be identified by size and their location is shown in (Figure 2A) [S3]. They are silent but respond with a burst of spikes to touch [S4]. The PlB is an extrinsic interneuron that inhibits the feeding network [S5]. It is a small neuron (20-30µm cell body diameter) that lies on the medial surface of the pleural ganglion (Figure 2A). It shows characteristic tonic firing activity and its identification is confirmed by recording inhibitory synaptic responses on feeding neurons. PeD12 is a newly discovered interneuron (60 µm in cell body diameter) that lies close to the previously described PeD11 [S6] on the dorsal surface of the pedal ganglia close to the statocyst (Figure 2A). Unlike PeD11, artificial stimulation of PeD12 excites PlB.

Intracellular dye-injection

To visualize the axonal branching of simultaneously recorded PlB and PeD12 cells, they were filled with the fluorescent dyes AlexaFluor 568 hydrazide (5 mM, Molecular Probes, Oregon, USA) and Lucifer Yellow dilithium salt (10 mM, Sigma, UK), respectively. The dyes were loaded into the cell bodies by a multi-channel picospritzer (General Valve Corporation, New Jersey, USA). After loading, the cells were incubated overnight in the fridge (4 C°). The isolated CNS was fixed with 4 %

paraformaldehidyde diluted in 0.1 M phosphate buffer (pH 7.4) for 1 hour at room temperature. The fixed CNS was placed on a cavity slide and it was mounted in Fluoroshield (F6182, Sigma). Carl Zeiss Laser Scanning Microscope (LSM) 510 was used to observe connections between the dye-filled cells.

Statistics

In both behavioral and *in vitro* experiments comparisons between independent groups were carried out using either t-tests or Mann-Whitney U tests. When comparing two paired groups where the distribution of data was not showing normality, tested with the Kolmogorov–Smirnov test the Wilcoxon signed-rank test was used. All statistical analyses were carried out using Prism (GraphPad Software). The data sets were considered statistically significant at $p < 0.05$. On the graphs error bars represent mean ± SEM.

Supplemental References

S1. Yeoman, M.S., Parish, D.C. and Benjamin, P.R. (1993). A cholinergic modulatory interneuron in the feeding system of the snail, *Lymnaea*. J. Neurophysiol. *70*, 37-50.

S2. Benjamin, P.R. and Rose, R.M. (1979). Central generation of bursting in the feeding system of the snail, *Lymnaea stagnalis*. J. Exp. Biol. *80*, 93-118.

S3. Ferguson, G.P., and Benjamin, P.R. (1991). The whole-body withdrawal response of *Lymnaea stagnalis*. II. Activation of central motoneurones and muscles by sensory input. J. Exp. Biol. *158*, 97-116.

S4. Ferguson, G.P. and Benjamin, P.R. (1991). The whole-body withdrawal response of *Lymnaea stagnalis*. I. Identification of central motoneurones and muscles. J. Exp. Biol. *158*, 63-95.

S5. Alania, M., Sakharov, D.A., and Elliott, C.J. (2004). Multilevel inhibition of feeding by a peptidergic pleural interneuron in the mollusc *Lymnaea stagnalis*. J. Comp. Physiol. A, *190*, 379-390.

S6. Syed, N.I. and Winlow, W. (1991). Coordination of locomotor and cardiorespiratory networks of *Lymnaea stagnalis* by a pair of identified interneurones. J. Exp. Biol. *158*, 37-62.
