# Supplementary material for: Interneuronal Mechanism for Tinbergen’s Hierarchical Model of Behavioral Choice
Source: Curr Biol. 2014 Sep 8;24(17):2018–24. doi: 10.1016/j.cub.2014.07.044 (PMC4159561; doi:10.1016/j.cub.2014.07.044)
Supplement: Document S2. Article plus Supplemental Information [file mmc2.pdf]

Current Biology 24, 2018–2024, September 8, 2014 ©2014 The Authors http://dx.doi.org/10.1016/j.cub.2014.07.044

Report

Interneuronal Mechanism for Tinbergen's Hierarchical Model of Behavioral Choice

Zsolt Pirger,[1,2] Michael Crossley,[1,2] Zita László,[1] Souvik Naskar,[1] György Kemenes,[1] Michael O'Shea,[1] Paul R. Benjamin,[1,3] and Ildikó Kemenes[1,3,*]
[1]Sussex Neuroscience, School of Life Sciences, University of Sussex, Brighton BN1 9QG, UK

Summary

Recent studies of behavioral choice support the notion that the decision to carry out one behavior rather than another depends on the reconfiguration of shared interneuronal networks [1]. We investigated another decision-making strategy, derived from the classical ethological literature [2, 3], which proposes that behavioral choice depends on competition between autonomous networks. According to this model, behavioral choice depends on inhibitory interactions between incompatible hierarchically organized behaviors. We provide evidence for this by investigating the interneuronal mechanisms mediating behavioral choice between two autonomous circuits that underlie whole-body withdrawal [4, 5] and feeding [6] in the pond snail *Lymnaea*. Whole-body withdrawal is a defensive reflex that is initiated by tactile contact with predators. As predicted by the hierarchical model, tactile stimuli that evoke whole-body withdrawal responses also inhibit ongoing feeding in the presence of feeding stimuli. By recording neurons from the feeding and withdrawal networks, we found no direct synaptic connections between the interneuronal and motoneuronal elements that generate the two behaviors. Instead, we discovered that behavioral choice depends on the interaction between two unique types of interneurons with asymmetrical synaptic connectivity that allows withdrawal to override feeding. One type of interneuron, the Pleuro-Buccal (PlB), is an extrinsic modulatory neuron of the feeding network that completely inhibits feeding when excited by touch-induced monosynaptic input from the second type of interneuron, Pedal-Dorsal12 (PeD12). PeD12 plays a critical role in behavioral choice by providing a synaptic pathway joining the two behavioral networks that underlies the competitive dominance of whole-body withdrawal over feeding.

Results

As predicted by the Tinbergen hierarchical model of behavioral choice [2], tactile stimuli that evoke whole-body withdrawal responses in *Lymnaea* significantly inhibit feeding even in the presence of a strong feeding stimulus (Figure 1). It should be noted that the animals (n = 16) were starved for 2 days so that they could be maximally sensitive to food stimuli.

[2]Co-first author
[3]Co-senior author
*Correspondence: i.kemenes@sussex.ac.uk
This is an open access article under the CC BY license (http://creativecommons.org/licenses/by/3.0/).

To investigate the interneuronal mechanisms underlying this behavioral choice, we developed a semi-intact preparation (Figure 2A) that allowed the application of tactile and sucrose stimuli to the lips while recording identified neurons from the feeding and withdrawal networks [7, 8]. We discovered that behavioral choice depends on the interaction between two unique types of interneurons. One type, the Pleuro-Buccal (PlB), is a well-known extrinsic feeding modulatory interneuron that has extensive inhibitory synaptic connections with interneurons and motoneurons of the feeding network [9]. The second type, Pedal-Dorsal12 (PeD12), is a newly discovered interneuron (Figures 2A and S1A available online) that plays a critical role in behavioral choice by providing the synaptic pathway that underlies the competitive interaction between the otherwise autonomous feeding and withdrawal-response networks.

Stimulation of PeD12 activated whole-body withdrawal and simultaneously inhibited rhythmic feeding movements (example in Figure 2B; n = 6). A burst of spikes (Figure 2B) artificially evoked in PeD12 by current injection resulted in a single large contraction of the columellar muscle, which is known to cause touch-induced whole-body withdrawal responses [4]. The same touch inhibited sucrose-driven rhythmic feeding movements of the buccal mass (feeding apparatus [10]). To understand how PeD12 might affect these alternative behaviors, we investigated the interactions of PeD12 with neurons of the withdrawal and feeding networks.

First, we asked how PeD12 drives withdrawal. We found that PeD12 is electrotonically coupled to motoneurons of the withdrawal-response network, and this plays a critical role in causing touch-induced withdrawal. A hyperpolarizing current pulse applied to PeD12 produced corresponding changes in membrane potentials of corecorded withdrawal motoneurons (Figure 2C1) that were located in several different ganglia of the CNS (Figure 2A). In the same preparation, application of lip touch caused a burst of spikes in PeD12 and motoneurons (Figure 2C2). Due to the extensive electrotonic connectivity of PeD12 with the withdrawal-response network, a current-induced burst of spikes in PeD12 depolarized the motoneurons and induced spiking (Figure 2C3) similar to that produced by touch. No other member of the withdrawal circuit was capable of eliciting withdrawal alone [5, 11]. It therefore seems reasonable to conclude that this behavioral response to touch results from a combination of distributed sensory input to all members of the withdrawal network [5] and the strong electrotonically mediated excitatory effects of PeD12 (Figure 2C3).

Next, we asked whether touch-induced burst responses in PeD12 are necessary for the touch-induced suppression of feeding in a sucrose-driven rhythm. Data supporting this necessity were obtained by recording PeD12 together with neurons of the feeding circuit, such as the B3 and B4 motoneurons (Figure 2A). By recording these motoneurons, we were able to monitor sucrose-driven "fictive feeding" activity, an in vitro correlate of behavioral feeding in the intact animal [10]. Motoneuronal bursts in response to sucrose were driven by synaptic inputs from the feeding central pattern generator (Figure 2D1, expanded trace). PeD12 was normally silent (mean resting potential −75 ± 2.3 mV, n = 28), but experiments

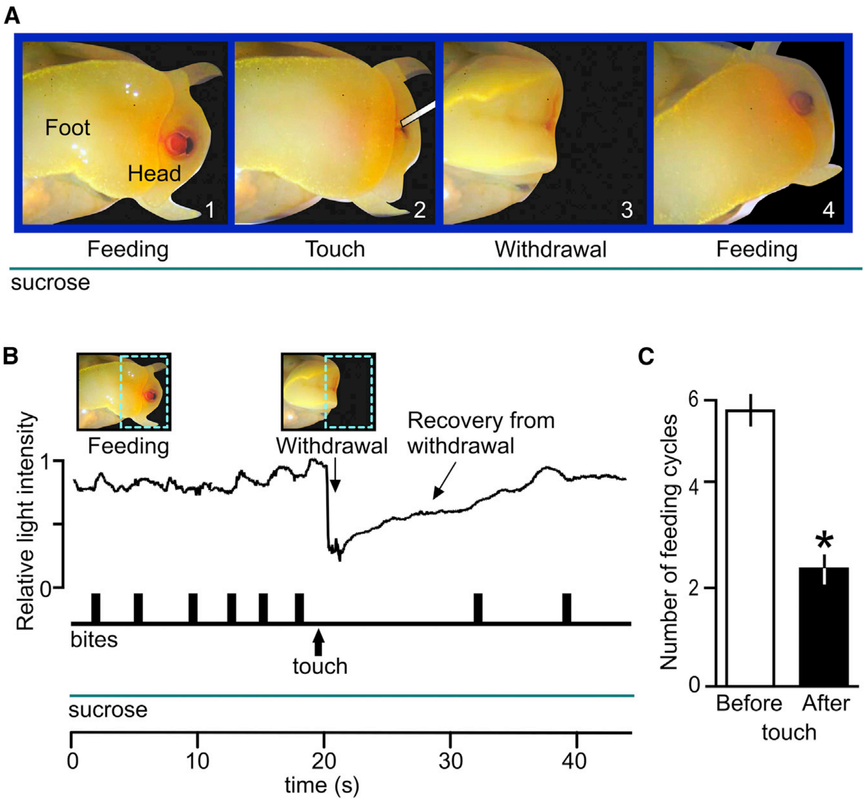

Figure 1. Behavioral Evidence for the Dominance of Touch-Induced Withdrawal over Feeding

(A) A video sequence of the head and anterior foot region showing feeding and withdrawal responses before and after the application of a touch stimulus. Sucrose is applied throughout the sequence. Feeding movements of the radula (bite) in response to food can be seen in frame 1, but these are inhibited by touch (mouth closed, frames 2 and 3). Touch also induces withdrawal responses in the head-foot region (frame 3). Frame 4 shows the resumption of feeding.

(B) Example of data obtained from the video recordings showing how the rhythmic feeding movements in response to continuous sucrose application are inhibited by a strong touch stimulus. Withdrawal responses are monitored by measuring the change in the relative light intensity caused by the retraction of the anterior head-foot region. The dashed region in the inserted image indicates the fixed sampling area for the measurement. The movement in response to a single touch consists of a rapid retraction, followed by a slower recovery phase when the head-foot returns to its original position. The recovery phase is probably passive because there are no known muscles that could account for this phase of the response. Individual bites that indicate the occurrence of an ingestive feeding cycle are shown as vertical bars in the lower trace.

(C) Six bites occur in the 20 s before touch, and two bites occur in the 20 s after touch. The number of feeding cycles in the 20 s before touch is significantly greater than the number in the 20 s after touch (n = 16; Wilcoxon signed-rank test: W = 136, $p < 0.0004$. Error bars show ±SEM).

(n = 12) of the type shown in Figures 2D1 and 2D4 showed that PeD12 was strongly activated by touch. This was accompanied by a significant inhibition of the fictive feeding rhythm recorded in the B3 feeding motoneuron. An artificially induced burst of spikes in PeD12 had the same effect (Figure 2D2). There was no statistical difference in the inhibitory effect on feeding between these two methods of spike activation (Figure 2D3). To determine whether PeD12 is necessary for feeding inhibition, we compared the effects of touch with (Figure 2D5) and without (Figure 2D4) suppression of touch-induced PeD12 spikes. Statistical analysis showed that preventing PeD12 spikes by hyperpolarization removed the inhibition of fictive feeding by touch (Figure 2D6). We conclude that the touch-induced spiking of PeD12 is necessary for inhibition of feeding.

We compared the effects of touch on feeding in the semi-intact preparation in which feeding was monitored in vitro with the behavioral experiments using the same stimuli (Figure 1). There was no significant difference in the inhibitory effects of touch in the two types of experiments, justifying the use of the in vitro preparations for the neural analysis of the Tinbergen choice mechanism (mean difference scores: behavioral, −3.1 ± 0.3, n = 16; in vitro, −2.5 ± 0.2, n = 17; Mann-Whitney test, U = 87, p = 0.07).

It was important to find out how PeD12 inhibited feeding because it was key to understanding how the two behavioral networks interacted. We found that there were no direct synaptic connections from PeD12 to neurons of the feeding network (Figure S2A). Instead, we showed that a PeD12 to PIB synaptic pathway mediated PeD12 inhibition of feeding, with PIB being the primary agent for feeding suppression. Evidence that PeD12 inhibited feeding via the PIB interneuron was obtained by corecording PeD12 and PIB and artificially manipulating their spike activity during a sucrose-driven rhythm (n = 3). Evoking a burst of spikes in PeD12 excited PIB, and this resulted in inhibition of feeding cycles recorded in the B3 motoneuron (Figure 3A1). Suppressing PIB activity by hyperpolarization prevented this inhibition (Figure 3A2), so PeD12 must have been acting via PIB. The ability of PIB alone to suppress feeding activity is shown in Figure 3A3, where a burst of spikes in PIB inhibited feeding in the absence of spike activity in PeD12.

These experiments suggest that PeD12 has an excitatory synaptic connection with PIB, and this was confirmed by showing that an artificially evoked burst of spikes in PeD12 drives an increase in the firing rate of PIB (Figure 3B1, left; n = 11). This connection was asymmetrical because there was no evidence of a corresponding synaptic connection from PIB to PeD12 (e.g., Figure 3B1, right) in the same preparation. More detailed experiments suggested that the PeD12-PIB synapse was chemically mediated and monosynaptic. Thus, calcium was required for transmission (n = 10) (Figure 3B2), and high concentrations of the divalent cations calcium and magnesium (Hi-Di saline), which blocked polysynaptic pathways [12, 13], did not block synaptic transmission (Figure 3B3, left; n = 8). When PIB spikes were suppressed by hyperpolarization in the same Hi-Di experiments, a slow depolarizing synaptic response was revealed (Figure 3B3, middle). Repeated triggering of single PeD12 spikes on a faster time base revealed the presence of short-latency 1:1 excitatory postsynaptic potentials (EPSPs) on PIB (Figure 3B3, right), also consistent with a monosynaptic connection. Dye-filling

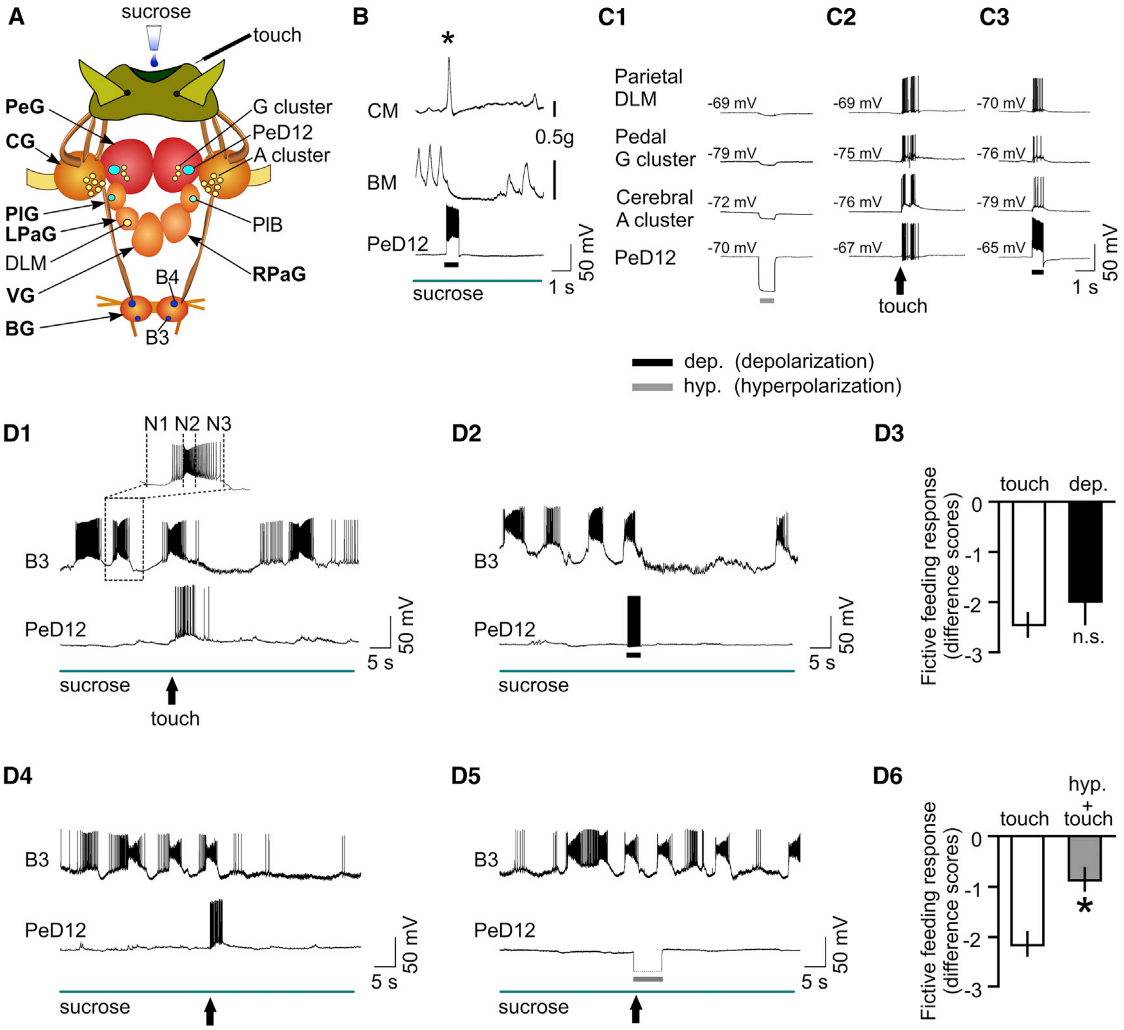

Figure 2. The Interneuron PeD12 Plays a Key Role in Behavioral Choice by Activating Withdrawal and Inhibiting Feeding in Response to Touch

(A) The semi-intact head-brain preparation used for recording interneurons and motoneurons of the feeding and withdrawal-response networks. This preparation retains the sensory nerves that carry touch and chemical signals from the lips to the central motor circuits. Paired PeD12 and PlB interneurons (light blue) are located in the pedal ganglia (PeG) and pleural ganglia (PlG), respectively. Feeding motoneurons, B3 and B4 (dark blue), are located in the buccal ganglia (BG). Motoneurons of the whole-body withdrawal network (yellow) are located in several CNS ganglia. The cerebral A cluster is the largest group (6–9 cells), with smaller numbers in the pedal G cluster (3–5 cells) and a single neuron (DLM) in the left parietal ganglion (LPaG). Other CNS ganglia are the right parietal ganglion (RPaG) and the visceral ganglion (VG).

(B) Responses to PeD12 stimulation recorded in the columellar muscle (CM) and the buccal mass (BM). The semi-intact preparation was used for these recordings, but for these experiments, the muscles involved in whole-body withdrawal (CM) and feeding ingestion (BM) were retained, and their contractions were recorded using a force transducer. Sucrose application drives rhythmic feeding movements in the BM until the evoking of a burst of spikes in PeD12 by current injection suppresses feeding despite the continued presence of sucrose. A single large contraction in the CM (*) is also caused by PeD12 stimulation (n = 6).

(C1–C3) Electrotonic coupling of PeD12 with motoneurons of the withdrawal-response network. Application of hyperpolarizing square current pulses to PeD12 causes similar but reduced responses in the three corecorded motoneurons (C1). Coupling coefficients recorded in the soma are 0.06 ± 0.01 (n = 5) between PeD12 and Parietal DLM motoneurons, 0.08 ± 0.1 (n = 5) between PeD12 and Pedal G cluster motoneurons, and 0.11 ± 0.02 (n = 12) between PeD12 and Cerebral A cluster motoneurons. Application of touch to the lips (C2) induces bursts of spikes in PeD12 and the three corecorded withdrawal-response motoneurons. A current-induced burst of spikes in PeD12 depolarizes the motoneurons and induces spiking in the motoneurons similar to that produced by touch (C3). All recordings shown in (C1)–(C3) are taken from the same preparation.

(D1–D6) Touch-induced spike activity in PeD12 is both sufficient and necessary for inhibition of feeding. The expanded trace of a B3 fictive feeding burst shows the N1 (protraction), N2 (rasp), and N3 (swallow) phases of the feeding cycle (D1). The inhibition of feeding by touch (D1) is similar to that induced by artificial stimulation of PeD12 (D2), and there is no statistical difference in the two types of data (D3) (n = 6, mean difference scores: touch, −2.4 ± 0.2; PeD12 depolarization, −2.0 ± 0.3; Wilcoxon signed-rank test, W = −8, p = 0.2). Hyperpolarizing PeD12 to suppress spiking (D5) during touch prevents the inhibition of feeding by touch (D4), producing a statistically significant reduction in the difference score (D6) (n = 9, mean difference scores: touch, −2.2 ± 0.2; PeD12 hyperpolarization, −0.9 ± 0.2; Wilcoxon signed-rank test, W = −36, p = 0.014).

In this figure and in the following figures, horizontal bars indicate that either a depolarizing (black) or a hyperpolarizing (gray) square current pulse has been applied. Difference scores in this and other figures are calculated by subtracting the number of feeding bursts in the 20 s before touch from the number of bursts in the 20 s after touch.

Error bars show ±SEM.

experiments revealed the sites of potential synaptic contacts between the two neurons (Figure S1). The arborization of PeD12 (Figure S1A1, red) and PIB (Figure S1A1, green) indicated two areas where the neurites intertwined. These were potential sites of the synaptic interactions. One of these areas was close to the cell body of the PeD12 cell (Figure S1A2), and the other was close to the PIB cell body (Figure S1A3). Together, these experiments provide evidence for a monosynaptic chemical pathway between PeD12 and PIB (Figure 3B4).

To validate the role of the PeD12-PIB synaptic pathway in behavioral choice, we had to show that PIB inhibits a sucrose-driven feeding rhythm. Although interneuron PIB inhibits feeding behavior [9], little is known about its sensory inputs [14], and in particular, about whether its response to strong tactile inputs is sufficient to suppress feeding rhythms. PIB fired tonically during sucrose application, but its baseline activity was insufficient to inhibit fictive feeding. A single touch stimulus produced a maintained depolarization of PIB and an increase in tonic firing (Figure 3C1). These touch-induced increases in PIB tonic firing rate resulted in an inhibition of the fictive feeding rhythm (Figures 3C1 and 3B1; n = 11). Similar inhibition was produced by an artificially evoked burst of spikes in PIB (Figure 3C2), indicating that increased firing in PIB was sufficient to suppress sucrose-induced feeding. A statistical comparison of the effects of touch versus the depolarization of PIB found that there was no difference in the inhibition of the fictive feeding responses produced by the two types of stimulation (Figure 3C3). The necessity for the touch-induced increase in firing of PIB for feeding inhibition was tested. PIB was hyperpolarized, and the effects of touch on fictive feeding were compared with (Figure 3D2) and without (Figure 3D1) hyperpolarization. There was a significantly smaller difference score in the hyperpolarized state (Figure 3D3). These results show that the increase in tonic firing in PIB induced by touch is both sufficient and necessary for the inhibition of feeding.

Finally, we showed that there were no synaptic connections between PIB and motoneurons of the withdrawal-response network (Figure S2B); therefore, PIB has no role in the control of whole-body withdrawal responses.

Discussion

Our results conform to the competitive model for behavioral selection originating in the ethological literature [2, 3] and provide an interneuronal mechanism for it. We propose that behavioral choice in response to conflicting sensory inputs depends on inhibitory synaptic interactions between autonomous networks that control incompatible behaviors. Extensive electrophysiological and anatomical investigations ([4–6]; Figure S2) show that the feeding and whole-body withdrawal circuits operate as autonomous units, consistent with the Tinbergen model. This type of inhibitory interaction between autonomous networks was suggested to occur in the mollusk *Pleurobranchaea* to explain the "dominance" of feeding over withdrawal [15, 16]. More recent studies [17, 18] have described the mechanism that mediates another type of competitive behavioral interaction in the same animal. Here, the dominance of swimming over feeding was shown to involve the asymmetrical synaptic inhibition of the feeding central pattern generator (CPG) circuit by a CPG interneuron from the swim circuit [17]. This differs from our example, where interneurons extrinsic to the feeding network are involved (Figure 4). The switch from feeding to defensive withdrawal is mediated by two identified interneurons with asymmetrical synaptic connectivity that allows withdrawal to always override feeding. One of these interneurons (PIB) completely inhibits the feeding rhythm when it is excited by the second of the two neurons (PeD12). Crucially, PeD12 also plays an important role in driving the whole-body withdrawal behavior, and it is responsive to strong tactile stimuli that evoke the defensive behavior. This pivotal neuron therefore has a dual function: in response to a strong aversive stimulus, it simultaneously activates the withdrawal motor circuit, acting as an extrinsic modulatory interneuron, and monosynaptically excites the PIB, which enhances tonic inhibition to the feeding motor circuit to suppress feeding. Thus, this simple asymmetric circuit joins the two motor networks and underlies the dominance of defensive withdrawal. By activating PeD12, the animal can simultaneously shut down grazing and initiate whole-body defensive withdrawal.

Defensive withdrawal of the whole animal is known to be at the top of the behavioral choice hierarchy in *Lymnaea* [19], so it would be expected that the inhibitory connection is asymmetrical to achieve this dominance. This differs from the original Tinbergen model, where reciprocal inhibition was proposed to prevent two behaviors being coexpressed. The reciprocal inhibition model is more likely to occur when inhibitory interactions between two autonomous behaviors require a more flexible relationship [20]. With high-value defensive behaviors, it is imperative that they override all other behaviors to prevent predation so that asymmetrical inhibitory interactions are present rather than reciprocal inhibition. In our experiments, we used starved animals to increase their responsiveness to food, but, despite this manipulation, food-driven feeding rhythms were still inhibited by touch. Tinbergen [2] considered that behavioral state would be an important determinant of behavioral selection, but our data suggest that aversive sensory stimulation triggering life-preserving behavioral responses overrides the effects of behavioral state.

Our *Lymnaea* example of behavioral choice is fundamentally different to other systems where the alternative behaviors share elements of one another's circuits. Here, behavioral choice depends on overlapping combinations of interneurons that are active during different behaviors. In the leech (*Hirudo*), for example, the selection of one of four different behaviors (swimming, shortening, crawling, or bending) depends on a unique combination of firing in the same interneurons [21].

A key feature of all those systems that involve network configuration is that similar groups of muscles and motoneurons are used in various combinations, so the units of motor control are not unique to a particular behavior. The Tinbergen model occurs when the elements of motor control are autonomous, and selection depends on hierarchically based control mechanisms, where behaviors are selected by inhibition of less-valued behaviors.

In conclusion, two distinctly different models have been proposed to explain how switching between behaviors is achieved at the level of neuronal networks. The selection of different combinations of active neurons in shared wider networks determines which of a limited subset of behaviors is expressed where there is significant overlap in the neuronal machinery and muscles controlling more than one behavior. The inhibition of one circuit by another determines the behavioral outcome when the choice is made between behaviors controlled by dedicated nonoverlapping networks. Our example is an interesting case of the second model. Moreover, it provides insight into the cellular and synaptic details of the way inhibition mediates behavioral choice. For example, our

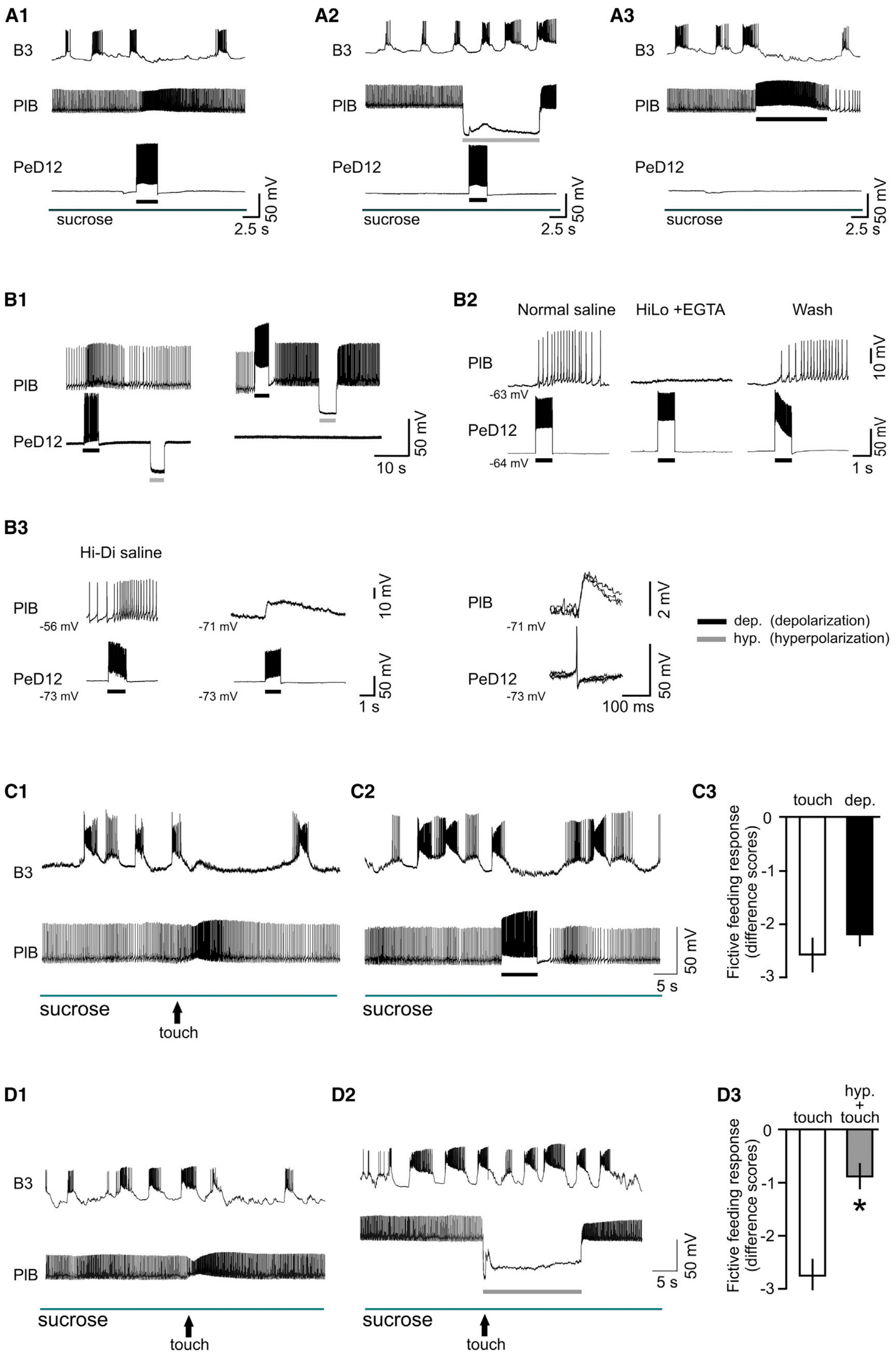

Figure 3. Monosynaptic Connection between PeD12 and PIB Mediates the Touch-Induced Inhibition of Feeding, and PIB is Both Sufficient and Necessary for Inhibition of a Sucrose-Driven Feeding Rhythm

(A1–A3) PeD12 inhibition of sucrose-driven fictive feeding is due to the excitation of the PIB interneuron (A1). A current-evoked burst of spikes in PeD12 increases tonic firing in the PIB interneuron and suppresses rhythmic bursting in the B3 motoneuron. The inhibition of the feeding pattern by PeD12 is

(legend continued on next page)

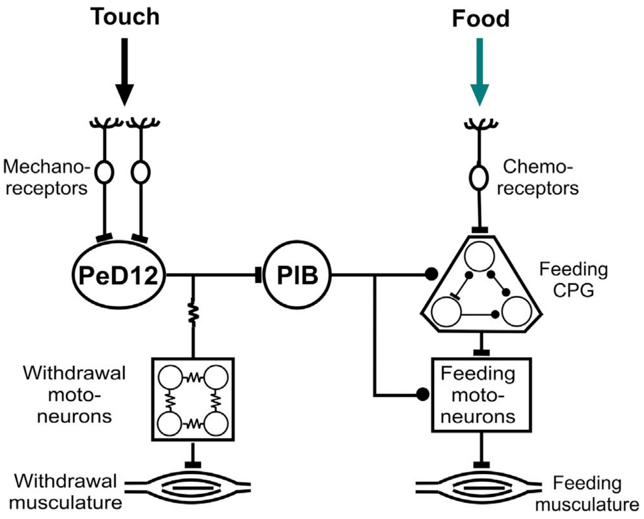

Figure 4. Summary Diagram of the Neuronal Mechanisms of Tinbergen-Type Decision Making in *Lymnaea*

Strong touch stimulation to the lips induces a burst of firing in PeD12, which in turn excites PIB via a monosynaptic excitatory connection. PIB inhibits the feeding network at multiple levels and stops ongoing feeding. However, PIB does not play a role in triggering withdrawal in response to touch. Thus, there is a clear asymmetry in the function of these two cells, with PeD12 coordinating activation of withdrawal with inhibition of feeding, whereas PIB is only involved in the inhibition of ongoing feeding in response to aversive touch. Bars indicate excitatory synaptic connection, and dots indicate inhibitory synaptic connections. Resistor symbols indicate electrotonic coupling between withdrawal-response motoneurons.

results suggest that there is an important role for the modulation of tonic inhibition in explaining the hierarchical coupling between behavioral responses to aversive and rewarding sensory stimuli. We therefore suggest that the regulation of tonic inhibition by interneurons constitutes a common mechanism that is central to adaptive behavioral switching in other systems [22, 23].

Experimental Procedures

Experimental Animals

Animals from a laboratory-bred stock of *Lymnaea stagnalis* were used in the experiment. Details of their maintenance are described in the Supplemental Experimental Procedures.

Behavior

Animals were starved for 2 days before the experiments. Sucrose-driven feeding activity was initiated by perfusion of 0.02 mM sucrose. Von Frey hairs (4 g) were used to induce whole-body withdrawal. The procedure was video recorded and analyzed using ImageJ software. Feeding scores were calculated by subtracting the number of feeding cycles in the 20 s after the touch from the number of cycles in the 20 s before.

Preparations

Experiments were performed on semi-intact preparations containing the entire CNS and attached lips and tentacles (Figure 2A) [8, 24–26]. A modified semi-intact preparation, containing the main feeding muscle (buccal mass) and the columellar muscle, responsible for the whole-body withdrawal was also used to measure contractions induced by neuronal stimulation. A detailed description of preparations, stimulation and recording protocols, explanation of choice of neurons recorded, and data analysis methods are described in the Supplemental Experimental Procedures.

Supplemental Information

Supplemental Information includes Supplemental Experimental Procedures and two figures and can be found with this article online at http://dx.doi.org/10.1016/j.cub.2014.07.044.

Author Contributions

Z.P. and M.C. carried out the electrophysiological experiments with the assistance of Z.L. M.C. carried out the behavioral experiments. S.N. did the confocal microscopy. G.K. and M.O. were involved in planning the experiments, discussing the results, and writing the manuscript. P.R.B. and I.K. each played a major role in the design of the experiments, analysis of the data, and production of the manuscript.

Acknowledgments

We would like to thank the Biotechnology and Biological Sciences Research Council for supporting our work.

Received: May 16, 2014
Revised: June 24, 2014
Accepted: July 16, 2014
Published: August 21, 2014

References

1. Kristan, W.B. (2008). Neuronal decision-making circuits. Curr. Biol. *18*, R928–R932.
2. Tinbergen, N. (1951). The Study of Instinct (New York: Oxford University Press).
3. McCleery, R. (1983). Interactions between activities. In Animal Behaviour, P.J.B. Slater and T.R. Halliday, eds. (Oxford: Blackwell), pp. 132–165.

prevented by hyperpolarizing PIB, showing that PeD12 acts via PIB (A2). Increasing tonic firing in PIB by current injection inhibits feeding without any spiking in PeD12 (A3; n = 3).

(B1–B3) Evidence for a chemically mediated monosynaptic connection between PeD12 and PIB. Stimulation of spike activity in PeD12 increases the firing rate of PIB. Hyperpolarizing pulses have no effect on PIB, suggesting that there is no electrotonic coupling (B1, right). Increasing the PIB firing rate by application of depolarizing current produces no response on PeD12 in the same experiment. Hyperpolarizing PIB also has no effect on PeD12 membrane potential or firing, so the synaptic connection between the two cells is asymmetrical. Perfusion of high magnesium/low calcium (Hi-Lo) EGTA saline in the semi-intact preparation blocks the PeD12-driven increase in PIB firing rate, which returns with washing in normal saline (B2). The Hi-Lo EGTA contains virtually no calcium and nine times the concentration of magnesium present in normal saline, and it blocks chemical synapses by replacing the calcium ions necessary for synaptic transmission with magnesium. Synaptic responses on PIB persist in high magnesium/high calcium (Hi-Di) saline, both at recorded membrane potential and when PIB is hyperpolarized to reveal a slow compound EPSP (B3). Triggering PeD12 spikes on a faster time base demonstrates that each superimposed spike results in a 1:1 unitary EPSP, indicative of a monosynaptic connection.

(C1–C3) Touch depolarizes PIB and increases tonic firing rate (C1). Statistical analysis showed that the membrane potential (MP) of PIB following touch stimulation was significantly depolarized compared with before (MP before: mean = −59.7 ± 1.6 mV; MP after: mean = −56.7 ± 1.9 mV, $p < 0.0002$, $t = 5.6$, degrees of freedom (df) = 10, n = 11) and that the firing rate was significantly increased as a result (before: mean = 2.8 ± 0.4; after: mean = 5.4 ± 0.4, $p < 0.0001$, $t = 8.6$, df = 10, n = 11). This results in the inhibition of feeding. This effect of touch is mimicked by artificially increasing PIB firing rates (C2). Statistical analysis shows that there is no difference in the inhibition of feeding using the two methods of PIB stimulation (C3) (n = 7, mean difference score: touch, −2.6 ± 0.3; PIB depolarization, −2.1 ± 0.1; Wilcoxon signed-rank test, W = −10, p = 0.3).

(D1–D3) Removing the excitatory effects of touch on PIB firing by hyperpolarization (D1) prevents the inhibition of feeding by touch (D2), and this is significant at the statistical level (D3) (n = 7, mean difference score: touch, −2.7 ± 0.4; PIB hyperpolarization, 0.9 ± 0.3; Wilcoxon signed-rank test, W = −28, $p < 0.02$). Error bars show ±SEM.

4. Ferguson, G.P., and Benjamin, P.R. (1991). The whole-body withdrawal response of *Lymnaea stagnalis*. II. activation of central motoneurones and muscles by sensory input. J. Exp. Biol. *158*, 97–116.
5. Ferguson, G.P., and Benjamin, P.R. (1991). The whole-body withdrawal response of *Lymnaea stagnalis*. I. identification of central motoneurones and muscles. J. Exp. Biol. *158*, 63–95.
6. Benjamin, P.R. (2012). Distributed network organization underlying feeding behavior in the mollusk *Lymnaea*. Neural Syst. Circuits *2*, 4.
7. Kemenes, I., Straub, V.A., Nikitin, E.S., Staras, K., O'Shea, M., Kemenes, G., and Benjamin, P.R. (2006). Role of delayed nonsynaptic neuronal plasticity in long-term associative memory. Curr. Biol. *16*, 1269–1279.
8. Marra, V., O'Shea, M., Benjamin, P.R., and Kemenes, I. (2013). Susceptibility of memory consolidation during lapses in recall. Nat. Commun. *4*, 1578.
9. Alania, M., Sakharov, D.A., and Elliott, C.J. (2004). Multilevel inhibition of feeding by a peptidergic pleural interneuron in the mollusc *Lymnaea stagnalis*. J. Comp. Physiol. A Neuroethol. Sens. Neural Behav. Physiol. *190*, 379–390.
10. Rose, R.M., and Benjamin, P.R. (1979). The relationship of the central motor pattern to the feeding cycle of *Lymnaea stagnalis*. J. Exp. Biol. *80*, 137–163.
11. Syed, N.I., and Winlow, W. (1991). Coordination of locomotor and cardiorespiratory networks of *Lymnaea stagnalis* by a pair of identified interneurones. J. Exp. Biol. *158*, 37–62.
12. Getting, P.A. (1981). Mechanisms of pattern generation underlying swimming in *Tritonia*. I. neuronal network formed by monosynaptic connections. J. Neurophysiol. *46*, 65–79.
13. Elliott, C.J., and Benjamin, P.R. (1989). Esophageal mechanoreceptors in the feeding system of the pond snail, *Lymnaea stagnalis*. J. Neurophysiol. *61*, 727–736.
14. Alania, M., Dyakonova, V., and Sakharov, D.A. (2004). Hyperpolarization by glucose of feeding-related neurons in snail. Acta Biol. Hung. *55*, 195–200.
15. Kovac, M.P., and Davis, W.J. (1977). Behavioral choice: neural mechanisms in *Pleurobranchaea*. Science *198*, 632–634.
16. Kovac, M.P., and Davis, W.J. (1980). Neural mechanism underlying behavioral choice in *Pleurobranchaea*. J. Neurophysiol. *43*, 469–487.
17. Jing, J., and Gillette, R. (1995). Neuronal elements that mediate escape swimming and suppress feeding behavior in the predatory sea slug *Pleurobranchaea*. J. Neurophysiol. *74*, 1900–1910.
18. Jing, J., and Gillette, R. (2000). Escape swim network interneurons have diverse roles in behavioral switching and putative arousal in *Pleurobranchaea*. J. Neurophysiol. *83*, 1346–1355.
19. Winlow, W., Moroz, L.L., and Syed, N.I. (1992). Mechanisms of behavioural selection in Lymnaea stagnalis. In Neurobiology of Motor Programme Selection, J. Kien, C.R. McCrohan, and W. Winlow, eds. (Oxford: Pergamon Press), pp. 52–72.
20. Anderson, D.J. (2012). Optogenetics, sex, and violence in the brain: implications for psychiatry. Biol. Psychiatry *71*, 1081–1089.
21. Kristan, W.B., and Gillette, R. (2007). Behavioural choice. In Invertebrate Neurobiology, G. North and R.J. Greenspan, eds. (Cold Spring Harbor: Cold Spring Harbor Laboratory Press), pp. 533–553.
22. Grillner, S., Hellgren, J., Ménard, A., Saitoh, K., and Wikström, M.A. (2005). Mechanisms for selection of basic motor programs—roles for the striatum and pallidum. Trends Neurosci. *28*, 364–370.
23. Benjamin, P.R., Staras, K., and Kemenes, G. (2010). What roles do tonic inhibition and disinhibition play in the control of motor programs? Front. Behav. Neurosci. *4*, 30.
24. Kemenes, G., Staras, K., and Benjamin, P.R. (1997). *In vitro* appetitive classical conditioning of the feeding response in the pond snail *Lymnaea stagnalis*. J. Neurophysiol. *78*, 2351–2362.
25. Staras, K., Kemenes, G., and Benjamin, P.R. (1999). Cellular traces of behavioral classical conditioning can be recorded at several specific sites in a simple nervous system. J. Neurosci. *19*, 347–357.
26. Kemenes, I., Kemenes, G., Andrew, R.J., Benjamin, P.R., and O'Shea, M. (2002). Critical time-window for NO-cGMP-dependent long-term memory formation after one-trial appetitive conditioning. J. Neurosci. *22*, 1414–1425.

Current Biology, Volume *24*
Supplemental Information

Interneuronal Mechanism for Tinbergen's Hierarchical Model of Behavioral Choice

Zsolt Pirger, Michael Crossley, Zita László, Souvik Naskar, György Kemenes, Michael O'Shea, Paul R. Benjamin, and Ildikó Kemenes

Supplemental figures

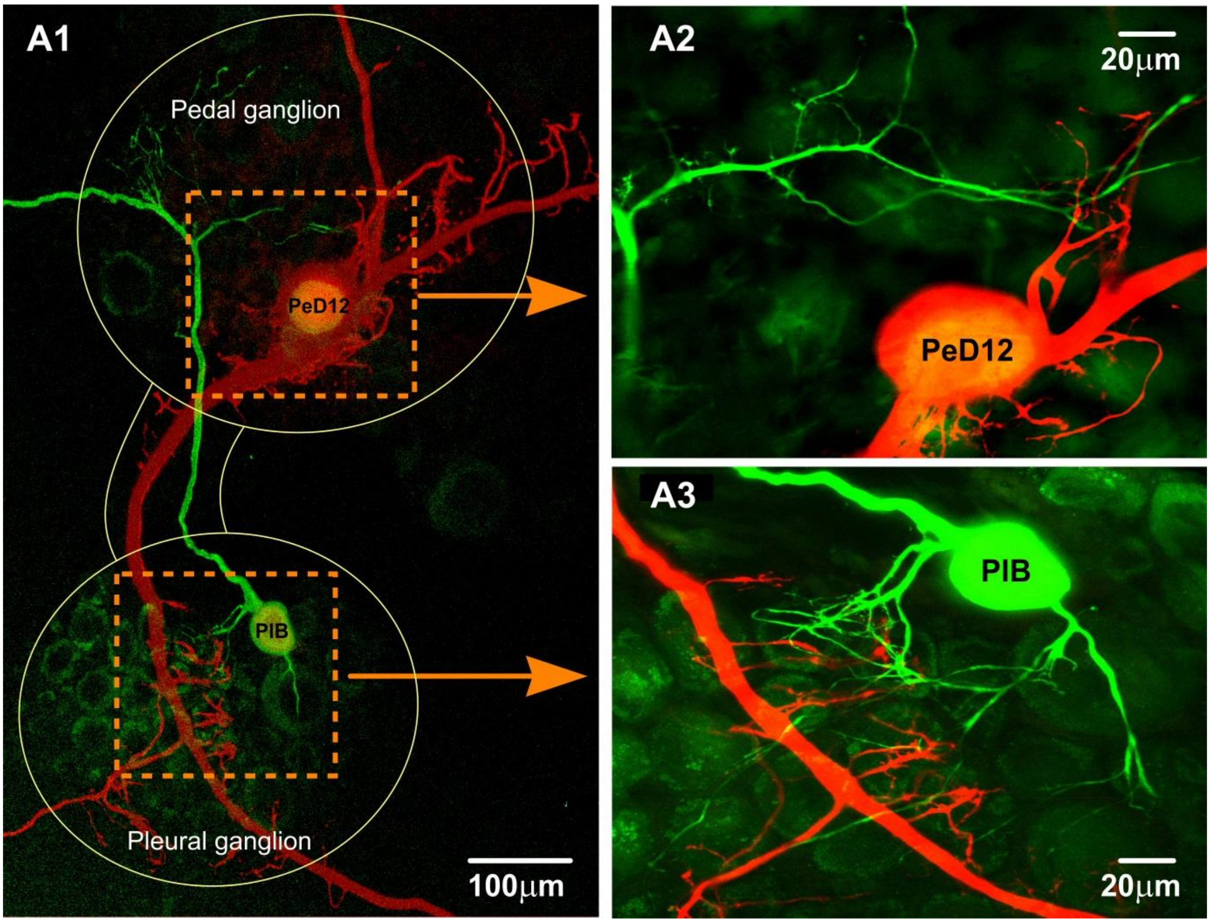

Figure S1. Morphology and Possible Sites of Synaptic Connections between the PlB and PeD12 Neurons.

(A1) Arborization and likely anatomical locations (dashed rectangles) of synaptic connections between the neurites of PeD12 and PlB, revealed by the injection of Alexa Red into PeD12 and Lucifer Yellow into PlB. (A2, A3). Two potential sites of the synaptic interactions recorded in the experiments shown in Figure 3A, B.

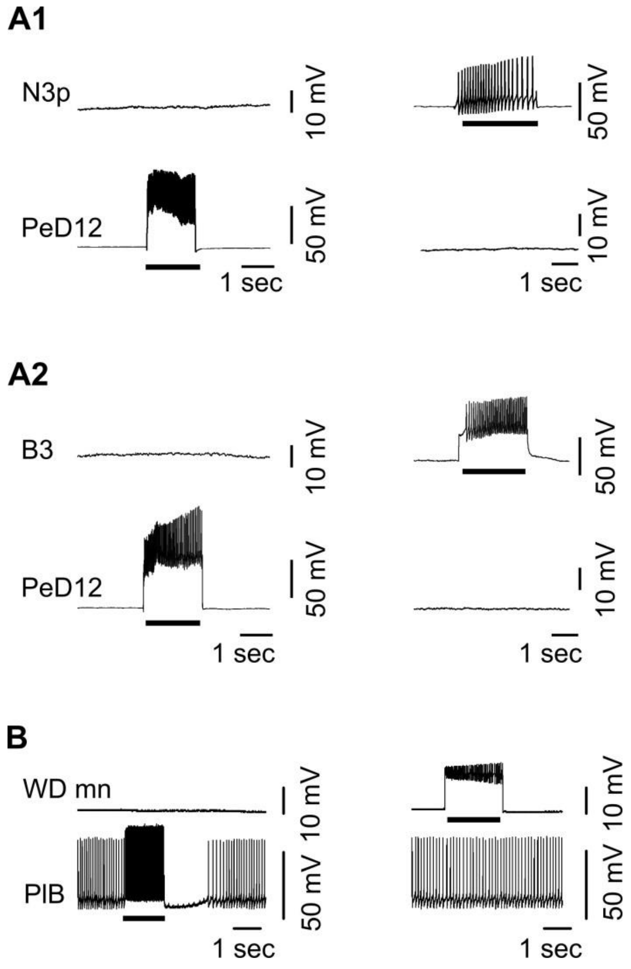

Figure S2. Lack of Direct Synaptic Connections between the Interneurons and Motoneurons that Control the Two Behavioral Circuits

There are no reciprocal synaptic connections between the PeD12 neuron and the central pattern generator interneuron, N3p (A1) and motoneurons (A2) of the feeding network (see Figure 2A, D). The experiments were carried out in Hi-Di saline to block any polysynaptic effects of PeD12 on feeding neurons. (B) There are no reciprocal synaptic connections between PIB and withdrawal motoneurons (WD mn) (Figure 2A, C).

Supplemental Data

Anatomical Evidence for Monosynaptic Connection between PeD12 and PlB

Injection of flourescent dyes revealed the likely location of the synaptic connection between PeD12 and PlB. Figure S1A shows the axonal projections of the two cells within the CNS ganglia. There is close intertwining of fine neuritic processes between PeD12 and PlB at two sites, one close to the PeD12 cell body in the pedal ganglion and the other close to the PlB cell body in the pleural ganglion (boxed areas). Viewing these sites at higher magnification shows the close apposition of the fine branching processes at these two locations, in the pedal (Figure SA2) and pleural (Figure SA1) ganglia neuropile. This combined with the electrophysiological data presented in Figure 3B suggest that the two interneurons are monosynaptically connected.

Lack of Direct Synaptic connections between PeD12 and PlB and Neurons of the Feeding and Withdrawal Response Networks

No reciprocal synaptic connections were found between PeD12 and central pattern generator interneurons such as the N3p (Figure S2A1) and motoneurons (Figure S2A2) of the feeding network (n = 12). No post-synaptic potentials or spike responses occurred as a result of current-evoked bursts of spikes in either cell types. These experiments were carried out in Hi-Di saline to prevent polysynaptic responses on feeding neurons due to the indirect PeD12-PlB pathway. There were a similar lack of reciprocal post-synaptic responses when PlB was recorded with motoneurons of the withdrawal response network (Figure S2B)(n= 10).

Supplemental Experimental Procedures

Animal Maintenance

Animals were kept in groups in large holding tanks containing copper-free water at 20°C on a 12:12h light-dark regime. The animals were fed lettuce three times and a vegetable based fish food (Tetra-Phyll; TETRA Werke, Melle, Germany) twice a week except before starting an experiment, when they were not fed for two days.

Supplemental Behavioral Procedures

To activate fictive feeding in the semi-intact preparations, sucrose (0.02 mM in normal saline) was applied to the lips via a computer controlled gravity-fed perfusion system. A mechanical probe connected to a DC power supply was used for tactile stimulation of the lips allowing for exact timing of touch stimulation. The stimulus, reliably generated whole body withdrawal responses monitored both on the columellar muscle and on the withdrawal motoneurons. The threshold level necessary for the whole-body withdrawal responses were carefully tested in both behavioral and semi-intact preparations to make sure we were dealing with the all–or–nothing responses rather than local graded responses. Experiments began 10 min after testing for a threshold. Preliminary experiments showed that keeping this time interval produces no change in the response. The buccal and columellar muscles were attached to force transducers (WPI FORT10g, World Precision Instruments, Incorporation, Sarasota, USA). Muscle recordings were made by connecting, the force transducers through a Digidata 1320A interface (Axon Instruments, Union City, CA, USA) to a PC.

Supplemental Electrophysiology Procedures

Preparations were dissected and recorded in a Sylgard-lined chamber containing normal snail saline (NS, 50 mM NaCl, 1.6 mM KCl, 3.5 mM $CaCl_2$, 2.0 mM $MgCl_2$, 10 mM HEPES, pH 7.9). The outer layer of the thick connective tissue sheath was removed mechanically from the dorsal surface of ganglia and the inner layers were softened by 1% protease treatment (Sigma XIV, Sigma) for 2 min. Synaptic connections between target cells were investigated in Hi-Lo EGTA saline (35.0 mM NaCl, 1.6 mM KCl, 18.0 mM $MgCl_2$, 10 mM HEPES, 2.0 mM EGTA, pH 7.9.) and Hi-Di saline (35.0 mM NaCl, 2 mM KCl, 14.0 mM $CaCl_2$, 8.0 mM $MgCl_2$, 10 mM HEPES, pH 7.9)[S1].

Intracellular recordings were performed under a fluorescence stereomicroscope (Leica MZ FLIII, Switzerland). AxoClamp 2B (Axon Instruments, Union City, CA, USA) and NeuroLog D.C. (Digitimer Ltd., UK) amplifiers were used to monitor the electrical activity of cells. Membrane potential manipulation was carried out by current injection through the recording electrode or by use of separate current-injecting and voltage–recording electrodes when more accurate recordings of membrane potential was required. Microelectrodes were pulled from borosilicate glass pipettes (GC200F-15, Harvard Apparatus, UK) with Narishige (Narishige Scientific Instrument Laboratory, Japan) vertical puller to a 18–24 MΩ tip resistance when filled with 4 M potassium acetate. For data acquisition and protocols, the amplifiers were connected via a DigiData 1320A interface (Axon Instruments, Union City, CA, USA) to a PC supplied with pClamp8.2 software (Axon Instruments, Union City, CA, USA). The recorded traces were analysed by OriginLab Corporation Origin8.5 software.

Identification of neurons

The B3 feeding motoneuron of the buccal ganglia was mainly used to monitor the CPG-driven fictive feeding rhythm. B4 feeding motoneurons were also routinely recorded to confirm the occurrence of feeding cycles. Both types of neuron can be identified by size, location and characteristic feeding activity [S2]. To monitor electrical activity underlying whole-body withdrawal, motoneurons of the cerebral A cluster, G cluster neurons of the pedal ganglion and the single DLM of left parietal were recorded, often in combination. These neurons can be identified by size and their location is shown in (Figure 2A) [S3]. They are silent but respond with a burst of spikes to touch [S4]. The PlB is an extrinsic interneuron that inhibits the feeding network [S5]. It is a small neuron (20-30µm cell body diameter) that lies on the medial surface of the pleural ganglion (Figure 2A). It shows characteristic tonic firing activity and its identification is confirmed by recording inhibitory synaptic responses on feeding neurons. PeD12 is a newly discovered interneuron (60 µm in cell body diameter) that lies close to the previously described PeD11 [S6] on the dorsal surface of the pedal ganglia close to the statocyst (Figure 2A). Unlike PeD11, artificial stimulation of PeD12 excites PlB.

Intracellular dye-injection

To visualize the axonal branching of simultaneously recorded PlB and PeD12 cells, they were filled with the fluorescent dyes AlexaFluor 568 hydrazide (5 mM, Molecular Probes, Oregon, USA) and Lucifer Yellow dilithium salt (10 mM, Sigma, UK), respectively. The dyes were loaded into the cell bodies by a multi-channel picospritzer (General Valve Corporation, New Jersey, USA). After loading, the cells were incubated overnight in the fridge (4 C°). The isolated CNS was fixed with 4 %

paraformaldehidyde diluted in 0.1 M phosphate buffer (pH 7.4) for 1 hour at room temperature. The fixed CNS was placed on a cavity slide and it was mounted in Fluoroshield (F6182, Sigma). Carl Zeiss Laser Scanning Microscope (LSM) 510 was used to observe connections between the dye-filled cells.

Statistics

In both behavioral and *in vitro* experiments comparisons between independent groups were carried out using either t-tests or Mann-Whitney U tests. When comparing two paired groups where the distribution of data was not showing normality, tested with the Kolmogorov–Smirnov test the Wilcoxon signed-rank test was used. All statistical analyses were carried out using Prism (GraphPad Software). The data sets were considered statistically significant at $p < 0.05$. On the graphs error bars represent mean ± SEM.

Supplemental References

S1. Yeoman, M.S., Parish, D.C. and Benjamin, P.R. (1993). A cholinergic modulatory interneuron in the feeding system of the snail, *Lymnaea*. J. Neurophysiol. *70*, 37-50.

S2. Benjamin, P.R. and Rose, R.M. (1979). Central generation of bursting in the feeding system of the snail, *Lymnaea stagnalis*. J. Exp. Biol. *80*, 93-118.

S3. Ferguson, G.P., and Benjamin, P.R. (1991). The whole-body withdrawal response of *Lymnaea stagnalis*. II. Activation of central motoneurones and muscles by sensory input. J. Exp. Biol. *158*, 97-116.

S4. Ferguson, G.P. and Benjamin, P.R. (1991). The whole-body withdrawal response of *Lymnaea stagnalis*. I. Identification of central motoneurones and muscles. J. Exp. Biol. *158*, 63-95.

S5. Alania, M., Sakharov, D.A., and Elliott, C.J. (2004). Multilevel inhibition of feeding by a peptidergic pleural interneuron in the mollusc *Lymnaea stagnalis*. J. Comp. Physiol. A, *190*, 379-390.

S6. Syed, N.I. and Winlow, W. (1991). Coordination of locomotor and cardiorespiratory networks of *Lymnaea stagnalis* by a pair of identified interneurones. J. Exp. Biol. *158*, 37-62.
